# Seabirds fighting for land: phenotypic consequences of breeding area constraints at a small remote archipelago

**DOI:** 10.1038/s41598-017-18808-7

**Published:** 2018-01-12

**Authors:** Guilherme Tavares Nunes, Sophie Bertrand, Leandro Bugoni

**Affiliations:** 1 Universidade Federal do Rio Grande (FURG), Laboratório de Aves Aquáticas e Tartarugas Marinhas, Instituto de Ciências Biológicas & Programa de Pós-Graduação em Oceanografia Biológica, CP 474 Rio Grande, RS Brazil; 2 Institut de Recherche pour le Développement (IRD), UMR248 MARBEC, Centre de Recherche Halieutique Méditerranéenne et Tropicale, Avenue Jean Monnet, BP 171, 34203 Sète Cedex, France; 3 Universidade Federal Rural de Pernambuco (UFRPE), Departamento de Pesca e Aquicultura, Departamento de Biometria, Rua Dom Manuel de Medeiros, s/n, Dois Irmãos, Recife, PE Brazil

## Abstract

Identifying associations between phenotypes and environmental parameters is crucial for understanding how natural selection acts at the individual level. In this context, genetically isolated populations can be useful models for identifying the forces selecting fitness-related traits. Here, we use a comprehensive dataset on a genetically and ecologically isolated population of the strictly marine bird, the brown booby *Sula leucogaster*, at the tropical and remote Saint Peter and Saint Paul Archipelago, mid-Atlantic Ocean, in order to detect phenotypic adjustments from interindividual differences in diet, foraging behaviour, and nest quality. For this, we took biometrics of all individuals of the colony breeding in 2014 and 2015 and tested their associations with nest quality, diet parameters, and foraging behaviour. While body size was not related to the foraging parameters, the body size of the females (responsible for nest acquisition and defence) was significantly associated with the nest quality, as larger females occupied high-quality nests. Our findings suggest that the small breeding area, rather than prey availability, is a limiting factor, emphasizing the role of on-land features in shaping phenotypic characteristics and fitness in land-dependent marine vertebrates.

## Introduction

Identifying associations between phenotypic variability and local selective pressures at the population level is crucial for understanding population differentiation and intraspecific diversity, along with predicting sensitivity to environmental changes^1,2^. Nonetheless, gene flow and genetic drift may hinder the role of local pressures at the population level, making it necessary to identify the patterns of the population structure and stochastic processes before tracking population-specific drivers of local adaptation^3,4^. In other words, for closely related populations with contemporary genotypes shared by gene flow, phenotypic diversity is adjusted as a balance between adaptation to local conditions and heritage from successful immigrants; so that, the fitness-related traits of genetically isolated populations are mostly dependent on local selective pressures^5^. Therefore, isolated populations are influenced by local conditions rather than inter-population cohesive forces (*e*.*g*., gene flow) and can even deviate from ecogeographical rules (*e*.*g*., Bergmann’s rule^6^), making them useful models for exploring the role of environmental features as selective forces for phenotype adjustment in wild populations^7^.

The effects of spatiotemporal environmental differences on phenotypic diversity have been extensively demonstrated in natural populations, which have been used as open air laboratories for understanding microevolutionary processes at the individual level^7,8^. Seminal research projects have demonstrated clear associations between morphological traits and environmental correlates, including the effect of predation pressure shaping colour of male guppies in South American streams^9^, the effect of rainfall and seed availability on bill size of Darwin’s finches from the Galapagos^10,11^, and the effect of the perch height and diameter selecting number of subdigital lamellae and leg size of *Anolis* lizards in Caribbean islands^12,13^. Currently, a variety of research tools have been applied to identify individual fitness-related traits and their environmental correlates in evolving natural populations from fungal prevalence and infection intensity^14^ to molar shape of mammals on islands^15^. However, information on the drivers of local adaptation in marine vertebrates is still scarce.

Field studies addressing evolution issues include important trade-offs that are often absent in laboratory studies due to the inherent complexities found in the natural environment^7^. Combining traditional and innovative techniques makes it possible to investigate natural selection, even in free-living animals, such as seabirds. Despite their high mobility, seabirds are known to contain population structure associated with colony-specific environmental conditions^16,17^, presenting gene flow disruption even between sympatric populations^18^. In this context, genetically isolated seabird populations arise as interesting models for testing individual quality and detecting fitness-related traits due to their high intraspecific diversity, high philopatry, colonial breeding, and dependence on resource availability around colonies^19^.

The brown booby *Sula leucogaster* is a strictly marine bird that breeds on oceanic islands and is distributed in tropical and subtropical regions of all ocean basins, exhibiting a strong phylogeographic structure^20,21^. Brown boobies form nests in small ground depressions and forage on prey available around colonies, so that specialization on local resources can disrupt gene flow and act as immigration filter for boobies from adjacent breeding sites^22^. This is suggested to be the case of a brown booby colony located in the remote and tropical Saint Peter and Saint Paul Archipelago (hereafter “SPSP”), an emerged portion of the Mid-Atlantic ridge. Boobies breeding at SPSP were demonstrated to be larger and heavier than the remaining brown booby colonies in the southwest Atlantic Ocean^6^, in addition to present a quite disrupted gene flow in relation to these colonies^22^. In this context, local adaptation was suggested to explain population isolation of brown boobies from SPSP, indicating that local selective pressures may be promoting individual specialization and population differentiation^22^.

Due to its geographical location and geological origin, SPSP is influenced by oceanographic conditions similar to seamounts, which spatially constrains prey distribution and limits the potential distribution of predators. This small-scale oceanographic dynamics increases local productivity and concentrates biomass around the archipelago, supplying a rich top predator community of resident species, such as bottlenose dolphins *Tursiops truncatus*^23^, brown boobies, black noddies *Anous minutus* and brown noddies *A*. *stolidus*^24^, and migratory species, such as whale sharks *Rhincodon typus*^25^, blackfin tunas *Thunnus atlanticus*^26^, and oilfish *Ruvettus pretiosus*^27^. Additionally, the small emerged area makes the configuration of the brown booby colony distinct from the typical pattern observed for this species. Colonies of brown boobies are usually small and comprised of scattered, irregularly spaced groups^28^, but the nest density of SPSP is unusually high due to the limited available area for nesting. Approximately 500 individuals breed in less than 0.006 km^2^ ^29,30^, and the resulting between-nest distance is only about 1 m^31^.

Given the environmental and population distinctiveness, we assessed the role of potential selective pressures in shaping phenotypes of brown boobies nesting at the SPSP. For this, we used an observational approach to extensively document the phenotypic variations, nest characteristics, diet, and foraging behaviour parameters. Our aim was primarily to identify current associations between the individual phenotypes and potential selective pressures rather than to describe population-specific microevolutionary processes, such as the strength and direction of selection, which require sampling over a longer time frame^1^. Genetic and phenotypic distances observed between brown boobies from SPSP and adjacent colonies (*i*.*e*., Fernando de Noronha Archipelago and Rocas Atoll) are suggested to be explained by local adaptations due to colony-specific selective pressures, highlighting the role of environmental features in isolating highly mobile top-predators^6,22^. Due to the comparatively large mean body size observed in brown boobies from SPSP^6^, we hypothesized that differences in the foraging parameters (*i*.*e*., diet and behaviour) and nest characteristics should be observed between the small and large individuals (*i*.*e*., a larger body size should be advantageous in some aspects in SPSP).

## Results

### Global results

**Figure 1:**
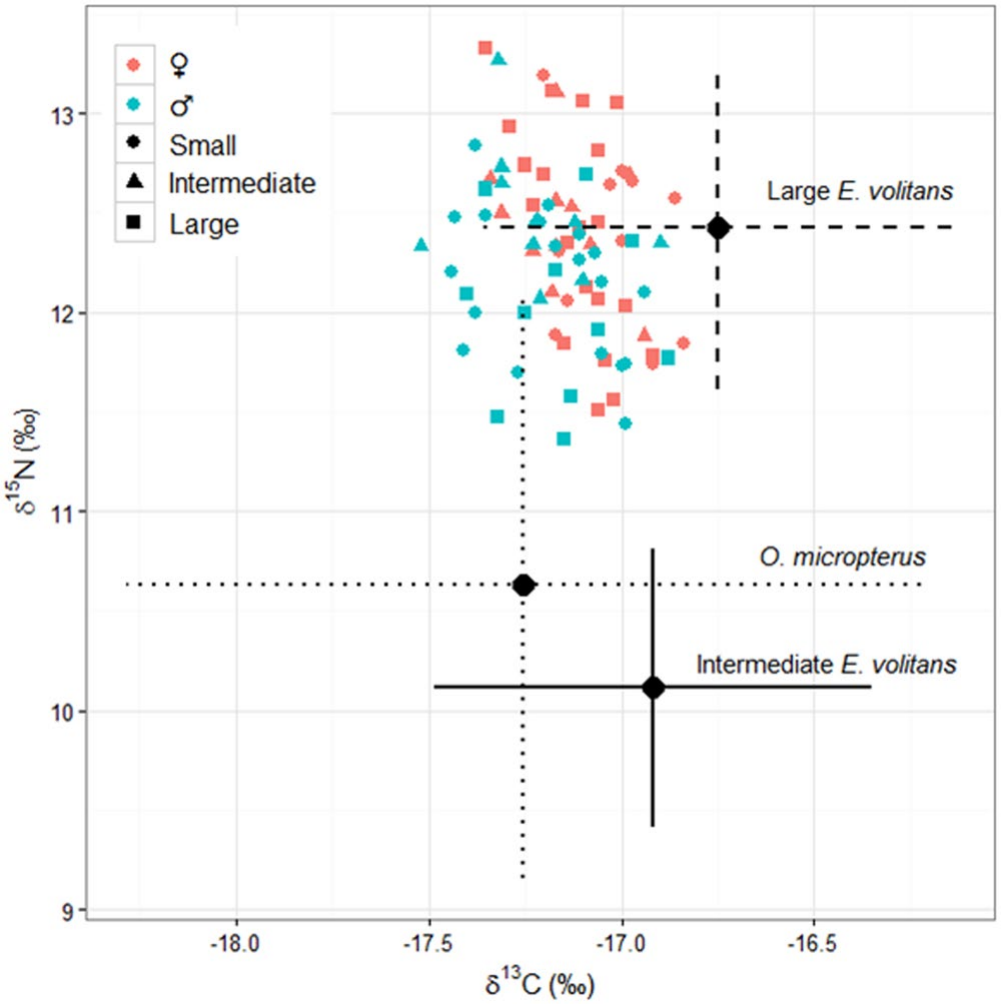
Isospace representing carbon (*δ*^13^C) and nitrogen (*δ*^15^N) isotopic ratios obtained in blood serum of brown boobies *Sula leucogaster* breeding in the Saint Peter and Saint Paul Archipelago and from muscle samples of flyingfishes. Brown boobies were grouped according to gender and body size. Prey items are: *Oxyporhamphus micropterus*; intermediate *Exocoetus volitans* = 100–150 mm fork length; and large *E*. *volitans* >150 mm fork length. Sampling was carried out in July 2015.

In total, 319 brown boobies (160 females and 159 males) had their biometrics assessed. On average, females were larger and heavier than males: 5.1% for culmen length, 7.1% for tarsus length, 4.4% for wing chord, and 25.3% for body mass (Table 1). Stable isotope values ranged from −17.52 to −16.82‰ for *δ*^13^C and from 11.24 to 13.33‰ for *δ*^15^N, and the isotopic standard ellipses areas (SEAc) were similar for males (0.22‰^2^) and females (0.18‰^2^). In total, 72 stomach contents and 307 individual prey from 60 birds (36 females and 24 males) were analysed. The asymptote of the prey species richness was reached with 21 stomach contents and 55 prey. All the seven prey items were identified to the species level, and the tropical two-wing flyingfish *Exocoetus volitans* (hereinafter referred to as ‘TTF’) was the most important prey item (PSIRI = 62.8%), followed by the bigwing halfbeak *Oxyporhamphus micropterus* (hereinafter referred to as ‘BH’; PSIRI = 17.6%) (Table 1; Supplementary Fig. 1). Stable isotopes measured from muscle samples of the prey species ranged from −18.28 to −17.31‰ for *δ*^13^C and from 7.42 to 11.11‰ for *δ*^15^N (Supplementary Table 1). Due to its high prey-specific importance, the TTFs were separated into three food item categories according to their fork length: small (<100 mm), intermediate (100−150 mm), and large individuals (>150 mm), for both the MixSIAR and subsequent %PSIRI analyses. Mixing models from MixSIAR using the three most important food items (large and intermediate TTF, and BH) as potential prey presented similar results: the mean posterior probabilities with a 95% credibility interval for females were 69.7% for the large TTFs, 7.2% for the intermediate TTFs, and 23.1% for the BHs, while for males they were 59.1%, 5.9%, and 35%, respectively (Fig. 1; Table 1; Supplementary Fig. 2).

**Table 1:** Data obtained (mean ± 1 standard deviation) from brown boobies *Sula leucogaster* breeding in the Saint Peter and Saint Paul Archipelago, in the Tropical Atlantic Ocean.

	♀				♂			
	Global	Small	Intermediate	Large	Global	Small	Intermediate	Large
**Phenotypic data**	(*n* = 160)	(*n* = 33)	(*n* = 32)	(*n* = 33)	(n = 159)	(*n* = 33)	(*n* = 33)	*n* = 33
Culmen length (mm)	108.2 ± 2.6	104.8 ± 1.8	107.3 ± 2.3	110.3 ± 1.7	103.2 ± 2.3	101.9 ± 2.1	102.83 ± 1.9	105.1 ± 1.9
Tarsus length (mm)	51.2 ± 1.9	48.3 ± 2.2	51.1 ± 0.9	52.8 ± 1.2	47.9 ± 1.4	45.9 ± 0.9	48.1 ± 0.9	49.2 ± 0.9
Wing chord (mm)	430.7 ± 7.4	423.1 ± 6.7	428.9 ± 6.8	435.8 ± 5.6	412.9 ± 7.2	405.4 ± 3.8	412.8 ± 5.6	418.5 ± 5.1
Body mass (g)	1834.5 ± 227.3	1581.3 ± 176.7	1777.1 ± 181.2	2032 ± 183.3	1545.5 188.9	1388.9 ± 190.2	1483.0 ± 154.6	1682.1 178.4
**Regurgitate material (%)**	(*n* = 44)	(*n* = 12)	(*n* = 7)	(*n* = 11)	(*n* = 27)	(*n* = 6)	(*n* = 2)	(*n* = 2)
*Exocoetus volitans*	66.2	79.6	90.7	80.6	59.3	57.4	41.6	76.3
*E*. *volitans* (large)	55.5	68.4	80.0	67.0	33.6	22.9	41.6	16.5
*E*. *volitans* (intermediate)	10.1	9.7	10.8	13.6	22.2	26.5	0.0	53.7
*E*. *volitans* (small)	0.6	1.5	0.0	0.0	3.5	8.0	0.0	6.0
*Hirundichthys affinis*	9.5	0.0	10.7	0.0	4.3	9.1	30.8	0.0
*Oxyporhamphus micropterus*	13.2	12.5	9.8	1.9	23.8	19.3	27.5	23.7
*Cheilopogon cyanopterus*	2.7	0.0	0.0	0.0	0.0	0.0	0.0	0.0
*Prognichthys gibbifrons*	4.3	0.0	0.0	3.9	11.9	7.6	0.0	0.0
*Euleptorhamphus velox*	2.9	0.0	0.0	9.7	1.5	6.6	0.0	0.0
*Ommastrephes bartramii*	2.5	0.0	0.5	3.8	0.9	0.0	0.0	0.0
**Stable isotopes (%)**	(*n* = 42)	(*n* = 11)	(*n* = 11)	(*n* = 20)	(*n* = 40)	(*n* = 19)	(*n* = 10)	(*n* = 11)
*E*. *volitans* (large)	87.3 ± 0.2	88.5 ± 0.2	87.5 ± 0.2	87.7 ± 0.2	75.3 ± 0.2	75.4 ± 0.2	80.1 ± 0.2	73.9 ± 0.2
*E*. *volitans* (intermediate)	3.1 ± 0.1	2.9 ± 0.1	2.9 ± 0.1	2.8 ± 0.1	3.2 ± 0.1	2.6 ± 0.1	2.0 ± 0.1	3.1 ± 0.1
*O*. *micropterus*	9.6 ± 0.2	8.6 ± 0.2	9.7 ± 0.2	9.5 ± 0.2	21.4 ± 0.2	21.9 ± 0.2	17.9 ± 0.2	23.0 ± 0.2
**Foraging behaviour**	(*n* = 52; 144 trips)	(*n* = 11; 62 trips)	(*n* = 14; 43 trips)	(*n* = 22; 42 trips)	(*n* = 45; 114 trips)	(*n* = 19; 57 trips)	(*n* = 10; 31 trips)	(*n* = 11; 34 trips)
Total trip duration (h)	0.9 ± 0.5	0.8 ± 0.4	0.9 ± 0.4	1.1 ± 0.6	0.9 ± 0.4	0.9 ± 0.4	0.8 ± 0.3	1.1 ± 0.4
Foraging range (km)	7.2 ± 3.9	6.8 ± 4.4	7.0 ± 2.7	7.7 ± 4.3	7.1 ± 4.8	6.5 ± 2.2	4.9 ± 2.5	8.4 ± 5.8
Distance covered (km)	27.0 ± 13.2	25.3 ± 13.5	24.4 ± 8.4	29.0 ± 15.4	27.1 ± 14.3	25.4 ± 11.5	21.1 ± 7.1	30.9 ± 14.2
Sinuosity	0.51 ± 0.1	0.49 ± 0.1	0.54 ± 0.1	0.52 ± 0.1	0.50 ± 0.1	0.50 ± 0.1	0.47 ± 0.2	0.50 ± 0.1
Flight speed (km.h^−1^)	29.4 ± 5.9	31.2 ± 3.5	29.4 ± 7.2	30.0 ± 5.4	28.1 ± 5.4	28.4 ± 4.7	25.8 ± 6.3	27.2 ± 4.3

Phenotypic data correspond to morphometrics and body mass. Diet contribution (%) was obtained from regurgitate material after calculating Prey-Specific Index of Relative Importance (%PSIRI) and from blood serum after stable isotope analyses (±95% credibility interval). Foraging behaviour was estimated by converting spatial data obtained with GPS dataloggers. ‘Global’ category also includes individuals not assigned for body size groups.

Foraging behaviour was recorded for 97 brown boobies (52 females and 45 males), summing to 258 trips. Mean (±1 standard deviation) trip duration was 57 ± 27.5 min, while mean maximum distance from the colony and the mean total trip length were 7.1 ± 4.3 km and 27.0 ± 13.7 km, respectively. Nest content had no significant effect on the foraging trip parameters and isotopic data for males or females; therefore, no individual was removed from the male and female datasets in subsequent analyses (Supplementary Fig. 3).

**Figure 2:**
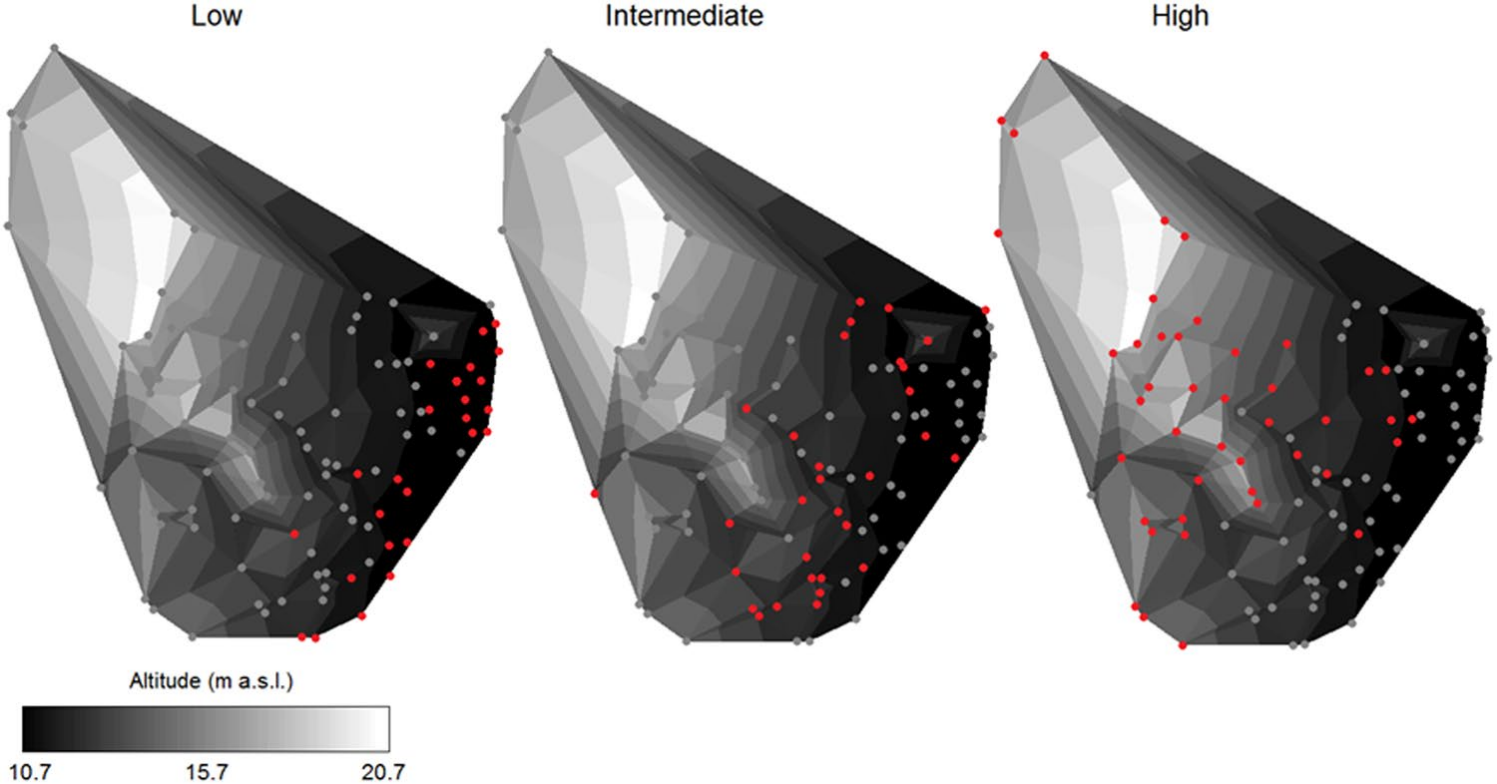
Locations of the 112 active nests (red points) of brown boobies *Sula leucogaster* in the Saint Peter and Saint Paul Archipelago in May–June 2014 and July 2015. Nests were classified as low, intermediate, and high quality, following a scheme based on colony features (Fig. 7). An interactive video of nest locations and island terrain is available as supplementary material.

In total, all the 304 nests were sampled for their landscape characteristics. Colony area was calculated using a minimum convex polygon and resulted in 601 m^2^, representing a density of 0.51 nests.m^−2^. Nest altitude ranged from 10.7 to 20.7 m a.s.l., and the average between-nest distance was 1.03 ± 0.16 m. Nest altitude was negatively correlated with the mean between-nest distance (*τ* = −0.15; *P* = 0.0004), meaning that the nest density was higher in the high-altitude areas. Using 15 m a.s.l. as a threshold, the nest density in areas >15 m was 0.89 nests.m^−2^ (114 nests in 128.9 m^2^), and it was 0.63 nests.m^−2^ (190 nests in 301 m^2^) in areas <15 m a.s.l. Although information for all the 304 active and non-active nests was used to build the scheme for nest classification, only the 112 active nests were considered in the subsequent analyses, which were 26.2% classified as low-quality, 29.9% as intermediate, and 43.9% as high-quality nests (Fig. 2).

### Intrasexual comparisons

**Figure 3:**
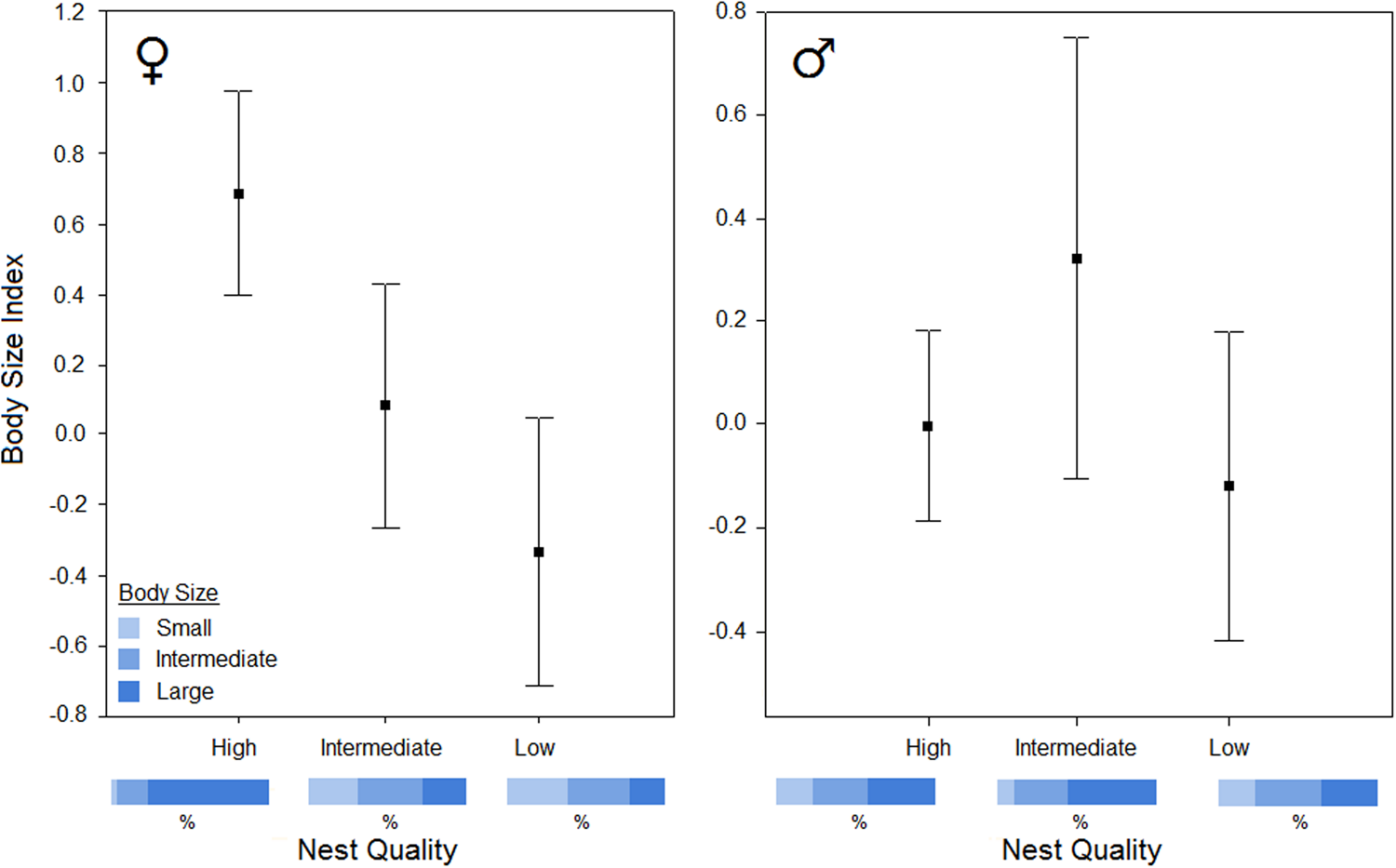
Distribution of body size of male and female brown boobies *Sula leucogaster* breeding in the Saint Peter and Saint Paul Archipelago for each category of nest quality, demonstrated by means ±95% confidence intervals error bars. Differences were significant (*P* < 0.001) only for females between high-low and high-intermediate nest quality. Body Size Index is the first principal component (PC1), which was calculated with standardized culmen length, tarsus length, wing chord, and body mass, and explained 66.1% of the variance. Blue bars are showing percentage of each body size group in each category of nest quality.

**Figure 4:**
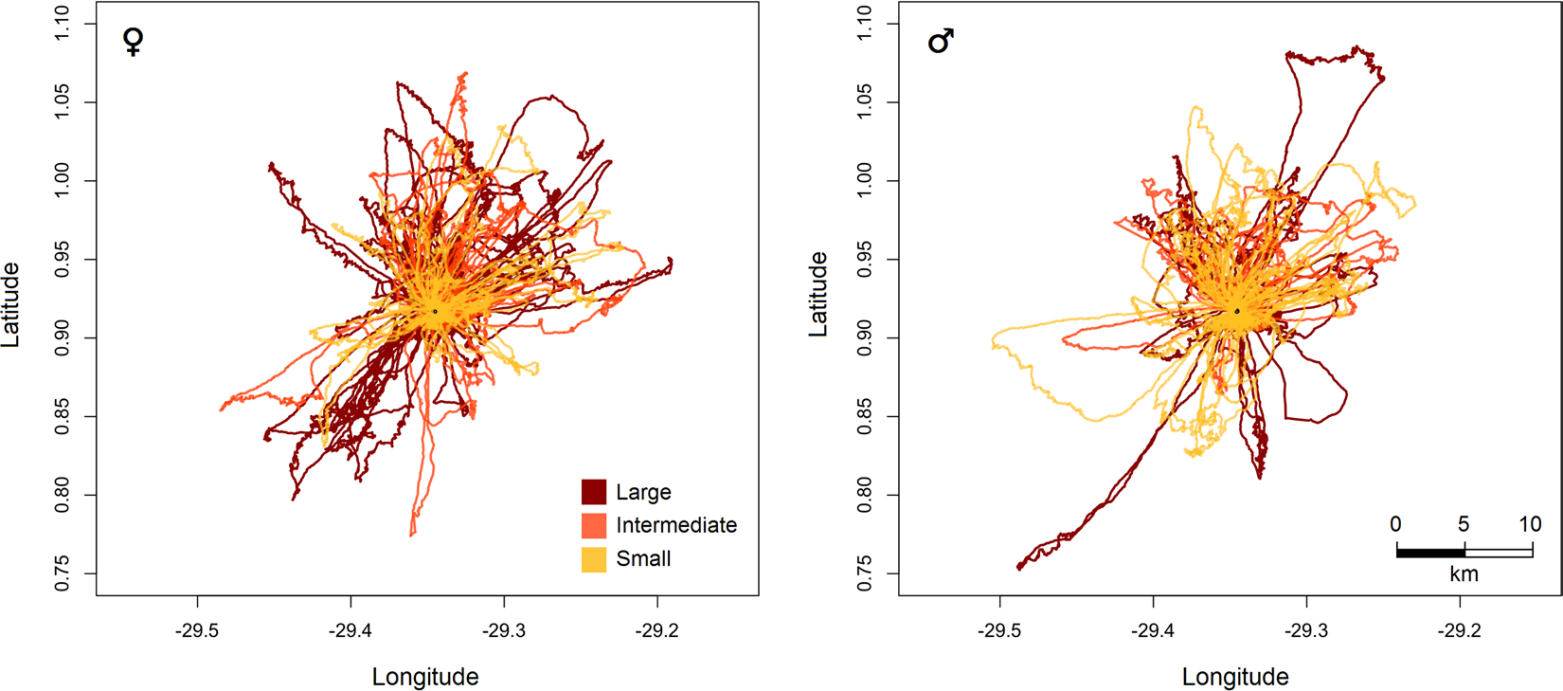
Foraging movements of 97 breeding brown boobies *Sula leucogaster* around the Saint Peter and Saint Paul Archipelago, recorded by GPS at 1 s and 10 s intervals, in July 2015. In total, 144 foraging trips from 52 females and 114 foraging trips from 45 males were recorded, of birds with large, intermediate and small body sizes.

**Figure 5:**
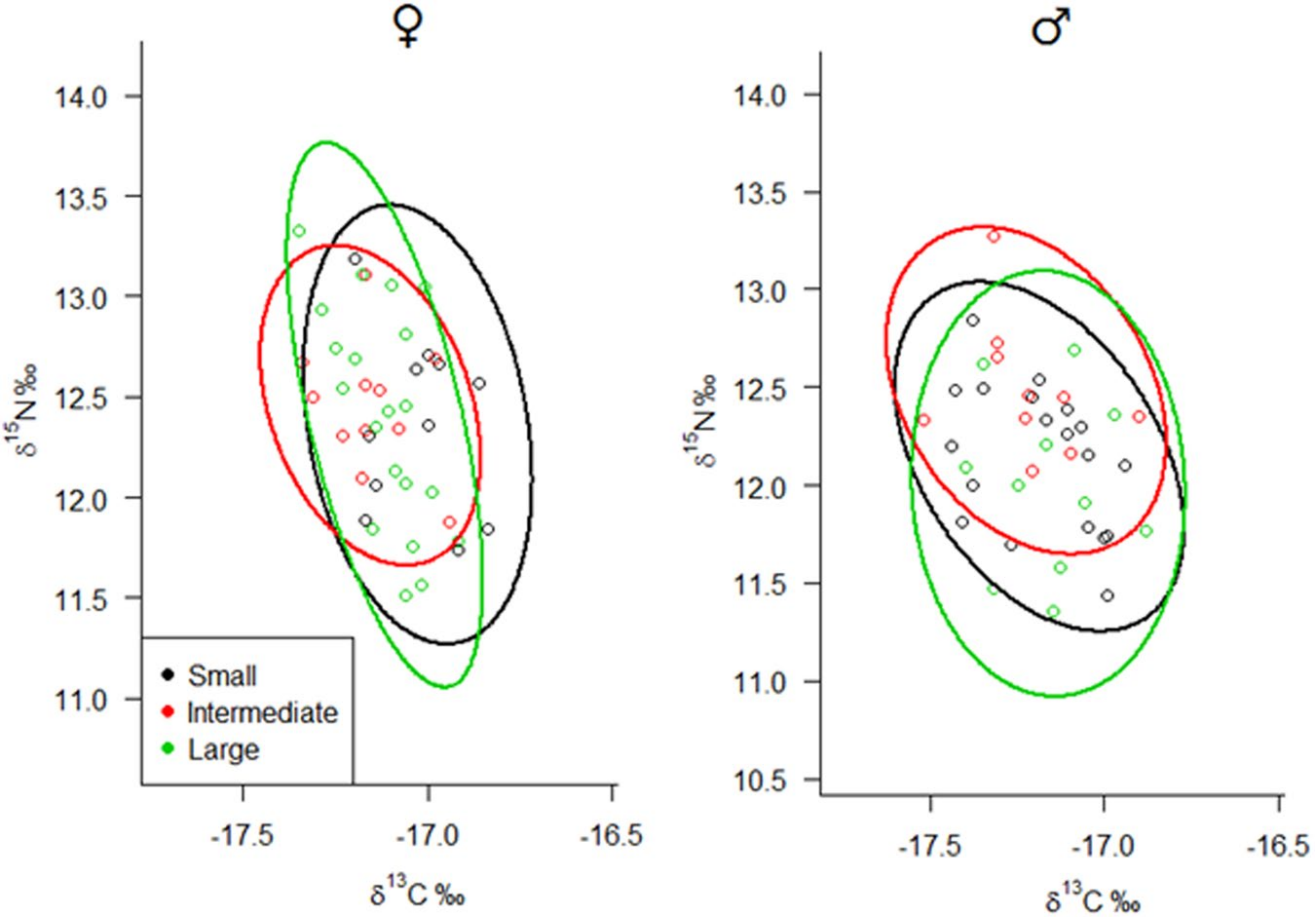
Standard Bayesian ellipses generated with carbon (*δ*^13^C) and nitrogen (*δ*^15^N) isotopic ratios from blood serum of brown boobies *Sula leucogaster* breeding in the Saint Peter and Saint Paul Archipelago, Brazil. Brown boobies were grouped according to gender and body size. Ellipses comprise 95% of the data.

Nest classifications were similar when comparing results from the hierarchical scheme and the scoring technique, so that 86% of the nests received the same classification in both approaches. Differences in the mean body size of females occupying nests of different quality were detected considering both the hierarchical scheme (*F*_*h*_ = 9.67; *P*_*h*_ = 0.0001) and the scoring technique (*F*_*s*_ = 5.749; *P*_*s*_ = 0.0043). Larger females predominated in high vs. low (*P*_*h*_ = 0.0002; *P*_*s*_ = 0.003) and high vs. intermediate (*P*_*h*_ = 0.02; *P*_*s*_ = 0.07) quality nests. In contrast, differences in the mean body size of males in relation to the nest quality were not detected (Fig. 3). Significant differences (*P* < 0.05) in the foraging trip parameters were not observed between the body size groups of males or females. In addition, significant correlation between the continuous body size index and trip parameters for either gender was not detected (Fig. 4; Table 1). In addition, no significant correlation or differences in the mean values were observed between the body size and *δ*^13^C and *δ*^15^N values, and the mean overlap of the Bayesian ellipses between the body size groups was 26% for females and 24% for males (Fig. 5). Prey item richness ranged from three to seven species among the body size groups of females and males, and TTF was identified as the food item of high prey-specific importance for both genders, corresponding to 79.6%, 90.7%, and 80.6% of the diet for the small, intermediate and large females and 57.4%, 41.7%, and 76.3% for the small, intermediate and large males, respectively (Table 1). There were no substantial differences in diet composition, inferred by the %PSIRI results, between the small, intermediate and large body sizes for the females or males, but comparisons between the %PSIRI results of the body size groups of the males had low power due to the small sample size (Table 1). Carbon and nitrogen isotopic ratios were statistically similar (*P* > 0.05) between the body size groups of males and females, and the contributions of potential food items generated by the mixing models were similar among groups (Fig. 1; Table 1). Finally, the highest importance of body size was observed in the regression trees explaining the variance in nitrogen for males (the third most important variable) and carbon for females (the second most important variable); the body size was not the most important variable in any regression tree (Supplementary Fig. 4).

## Discussion

Significant differences in the mean body size were detected between the females nesting in low and high-quality nests, but associations of diet and foraging trip parameters with body size of both genders were not observed. Brown boobies breeding in SPSP have marked intersexual differences in nest attendance so that females dedicate significantly more time to incubation, brooding, and chick provisioning^31^. Furthermore, nest defence is also female-biased in boobies from SPSP^31^ and can be critical to ensure egg or chick safety and avoid extreme injuries, such as chick predation by cannibalism^32^. Considering the high colony density found in SPSP, we suggest that spatial constraints for nesting are the major driver for shaping fitness traits in brown boobies, positively selecting large females, which would benefit from their body size during fights for acquisition and defence of high-quality territories for nesting.

Interestingly, the normal distribution curve of female body size was more acute than that of males, but slightly skewed towards the large females, suggesting decreased phenotypic variability and reinforcing the idea of positive selection acting on intermediate and large females. The parameters we used to classify the quality of nests are closely related to breeding success in other colonial bird species^33–35^, and therefore, the predominance of small females in low-quality nests suggests a negative selection on this group. Indeed, mean body size and mass of brown boobies from SPSP are not explained by ecogeographical rules, such as the Bergmann’s rule, and are the highest among brown boobies breeding along the southwest Atlantic Ocean^6^, suggesting higher breeding success of the large and intermediate individuals in comparison to small ones, and increasing the average body size of birds in this population. Furthermore, the population is genetically distant from adjacent populations (*i*.*e*., Rocas Atoll and Fernando de Noronha Archipelago), which could be explained by an adaptation to the local seascape, causing phenotypic differentiation and, consequently, selection against immigrants^22^.

Absence of intrasexual differences in foraging behaviour could be associated with the abundant food around SPSP. Brown boobies from the Gulf of California spent ~2.5 h at sea during foraging trips and travelled almost 80 km on average, flying up to 28 km from the colony^36^. In the Caribbean, brown boobies spent ~6 h at sea during foraging trips, travelling an average of 129 km, up to 48 km from the colony^37^. The relatively small foraging range of brown boobies around SPSP, revealed by the short mean trip duration (57 min), mean maximum total distance (27 km), and maximum distance from the colony (7 km), suggests there is plenty of food available in the immediate surroundings of the archipelago. This is consistent with the seamount-like oceanographic dynamics observed around SPSP, which enhances local primary productivity on a very small spatial scale due to the interaction of the Equatorial Undercurrent with the submarine relief, increasing the residence time of nutrients and generating vortices and small-scale upwellings in the surroundings of the archipelago^38,39^. Though individual differences in foraging trip parameters in relation to the body size have been demonstrated for other booby species^40,41^, prey seems to be easily accessible for all boobies at SPSP, and thus obtaining food does not seem to be the main selection pressure on the phenotypes of boobies from SPSP.

Exocoetidae flyingfishes represented more than 95% of the material regurgitated by brown boobies as a whole, emphasizing the importance of this family to sustain a diverse epipelagic community around a tropical remote archipelago. Flyingfishes play a central role in making SPSP an important site for the tropical Atlantic Ocean epipelagic food web, as they spawn around the archipelago and represent approximately 80% of the fish larvae found in its surroundings during the northern summer, of which 73% is composed by the margined flyingfish, *Cheilopogon cyanopterus*^42,43^. Interestingly, the margined flyingfish, a 330 mm-length species^44^, has been identified as the most important prey species for wahoo *Acanthocybium solandri*, yellowfin tuna *Thunnus albacares*^45^, oilfish^46^ and brown booby^47^ in the SPSP, but in this study, it was not present in the regurgitated material of males and was only rarely found in the female diet. In turn, TTF is not considered to be an important prey for these top predators, and BH appears to be secondary in importance for seabirds; however, in our study, TTF was dominant in the regurgitated material of both male and female brown boobies. The margined flyingfish is abundant around SPSP from November to April, when it is believed to spawn and use rocks for egg mass attachment^44^. Indeed, the highest capture rates of pelagic fishes around SPSP are during this period, mainly through the exploitation of yellowfin tuna, which are attracted to the high concentrations of margined flyingfish, while the lowest capture rates are observed during the northern winter, when this study was carried out^45,46^. Therefore, we suggest that flyingfishes support breeding activities of brown boobies in SPSP, but the main prey species appears to alternate between margined flyingfish during the southern summer^47^ and TTF during the southern winter (as found here), demonstrating the consequences of the high primary productivity on the food web in SPSP throughout the year, despite this being a tropical remote archipelago surrounded by oligotrophic waters.

Studies addressing fine individual differences could benefit from research tools with even higher resolution and information provided by long-term monitoring programmes. We are convinced that there are individual covariates influencing brown boobies nesting at SPSP, such as personality^48^, senescence^49^, and early body condition^50^, which could influence intrapopulational parameters. Additionally, dataloggers capable of detecting behaviour at a higher resolution, such as accelerometers and time-depth recorders, could provide valuable information on additional attributes, such as underwater activities^51^ and daily energy expenditure^52^. Monitoring breeding success of small, intermediate and large individuals, as well as of females using low, intermediate and high-quality nests is crucial to understand how the limited breeding area is determining fitness of brown boobies at SPSP. The relatively long breeding cycle and the logistics to stay at the colony make it difficult, but remote monitoring could be carried out by using cameras, for example.

Nonetheless, our findings demonstrate that the available area in terrestrial environments could be a relevant limiting factor even for strictly marine vertebrates, such as seabirds. This is particularly relevant for seabird conservation, as habitat degradation and human disturbance of colonies have been attributed as major threats to seabirds globally^53^; thus, decreasing the area available for nesting could make territory disputes fiercer and influence which individuals will occupy high-quality nests. Other marine vertebrate species that depend on land for reproductive activities could also be affected by the individual ability to obtain high quality breeding locations. For example, it has been reported that grey seals *Halichoerus grypus* breeding on sand dunes do not have breeding site fidelity due to the changes and unpredictability of colony landscape^54^, but grey seals breeding on rocky islands have shown colony and breeding location fidelity, suggesting that colony landscape heterogeneity promotes interindividual disputes for high-quality breeding areas^55^. Additionally, climate change can strongly affect ice-dependent species by depleting the ice conditions required for breeding, increasing youth mortality and the frequency of sabbatical years, and forcing dispersal to more suitable breeding areas^56,57^. Over the past 100 years, the global mean sea level has risen by 0.19 m and is expected to increase ~0.5 m by 2100 compared to the current level^58^. Based on this estimate, the breeding activities of boobies nesting in the lowest areas at SPSP will be even more difficult than they currently are, possibly resulting in population declines due to both the reduction of suitable nesting areas and a trend of increasing average body size due to the intensification of disputes for high-quality nests. In this context, understanding current limiting factors, individual quality and population plasticity is crucial to predict how natural populations will cope with rapid environmental changes promoted by human disturbance in colonies, such as habitat degradation and global warming.

## Material and Methods

### Study area

**Figure 6:**
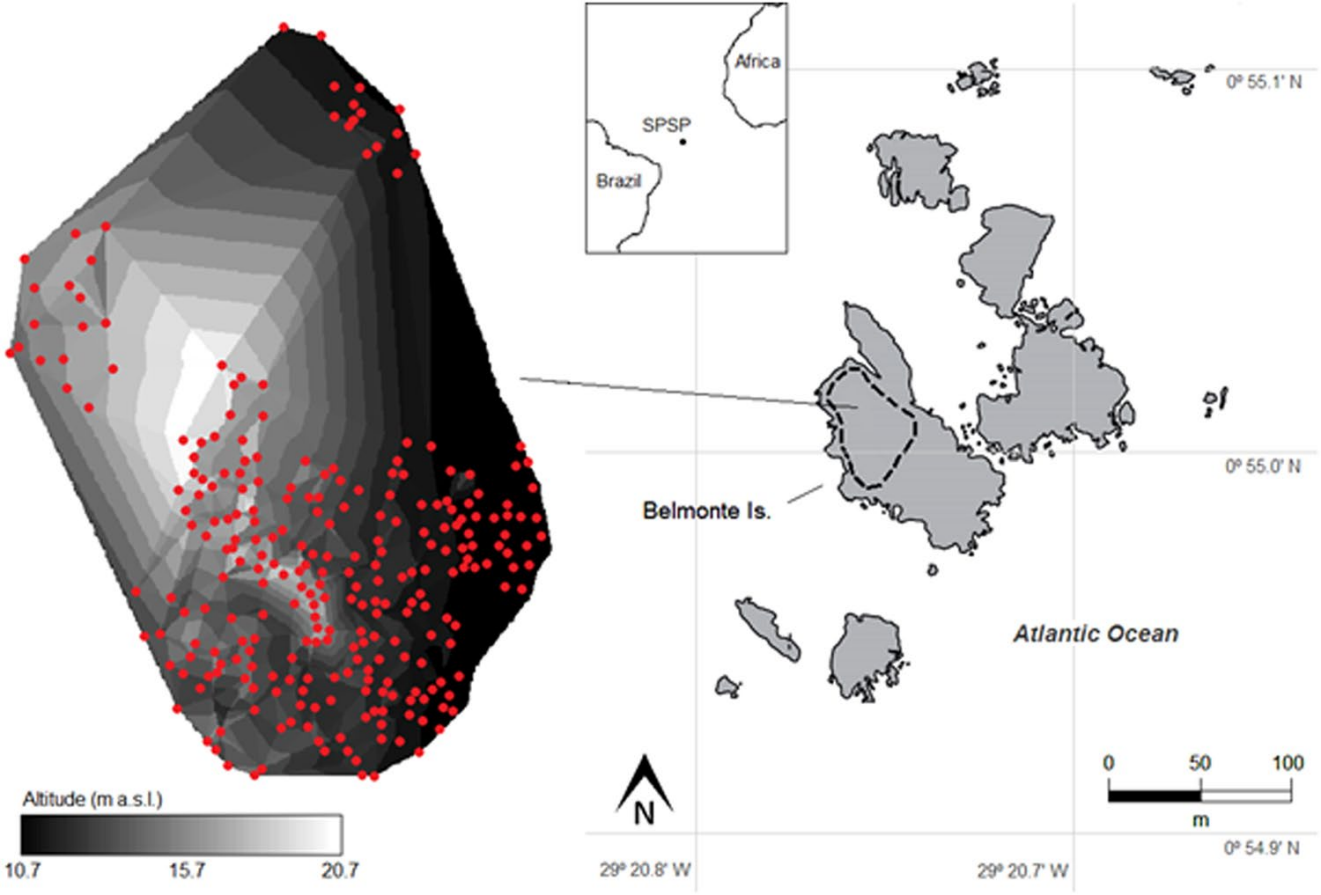
Schematic map of the study area in the Saint Peter and Saint Paul Archipelago. Sampling was carried out in the Belmonte Island, where there is a dense colony of brown boobies *Sula leucogaster* with breeding activities throughout the year (highlighted area). All the 304 active and non-active nests (red points) of the Belmonte Island were sampled for latitude, longitude, and altitude with 20 mm-resolution, which were used to build isolines of altitude for the colony. For a 3D view of nests see Supplementary Video. The map was modified from Barbosa-Filho & Vooren (2010^29^); CCBY Attribution license: http://www4.museu-goeldi.br/revistabrornito/revista/index.php/BJO/index.

Saint Peter and Saint Paul is a small remote tropical archipelago composed of ten islets with a total area of 0.017 km^2^ and located at 0°55′N and 29°20′W, on the mid-Atlantic ridge. The archipelago holds colonies of brown noddies, black noddies, and brown boobies, the latter breeding mainly on Belmonte Island, which has a maximum altitude of 21 m and an area of 6000 m^2^ ^30^. Despite the spatial constraint, the brown booby colony on Belmonte Island has over 100 nests this study^31^, which are distributed on the sloping stones located on the northwest face of the island (Fig. 6). The colony is heterogeneous regarding landscape features; for example, the nests vary in altitude, distance from the sea, susceptibility to waves, and between-nest distances. As SPSP is located on the Equator, it is directly influenced by the seasonality of the Intertropical Convergence Zone, a low-pressure area that meets easterly surface winds forming a cloudy band with increased precipitation around the globe^59^. From February to May, the Intertropical Convergence Zone is between 7°S and 8°N and by April, the maximum accumulated precipitation reaches 370 mm on SPSP^38^. Furthermore, SPSP has a seamount-like seascape in its surroundings and is influenced primarily by the Equatorial Undercurrent, which flows eastward at a depth of ~80 m, slowing when it reaches the archipelago, generating vortices and small-scale upwelling that increase local primary productivity^38,60^.

### Nest characterization

All the active and non-active nests (*i*.*e*., those only containing nest material such as small stones and feathers) were georeferenced using the Real-Time Kinematic (RTK) method with a preprocessed base point and a rover station (Topcon Positioning Systems, Inc.), which was placed at the central point of each nest. Non-active nests are also easily identified by the configuration of the nest material, making it possible to locate the centre of the nest to take the point. Radio-based communication between base and rover stations provided real-time corrections when triangulating to GPS satellites so that approximately 20 mm-resolution latitude, longitude, and altitude data were obtained for each nest. After recording data for 304 points (*i*.*e*., all the active and non-active nests) in 1580 m^2^, a three-dimensional elevation map was built with a resolution of 0.19 points.m^−2^. Additionally, the nests were individually numbered using photographs of the colony.

**Figure 7:**
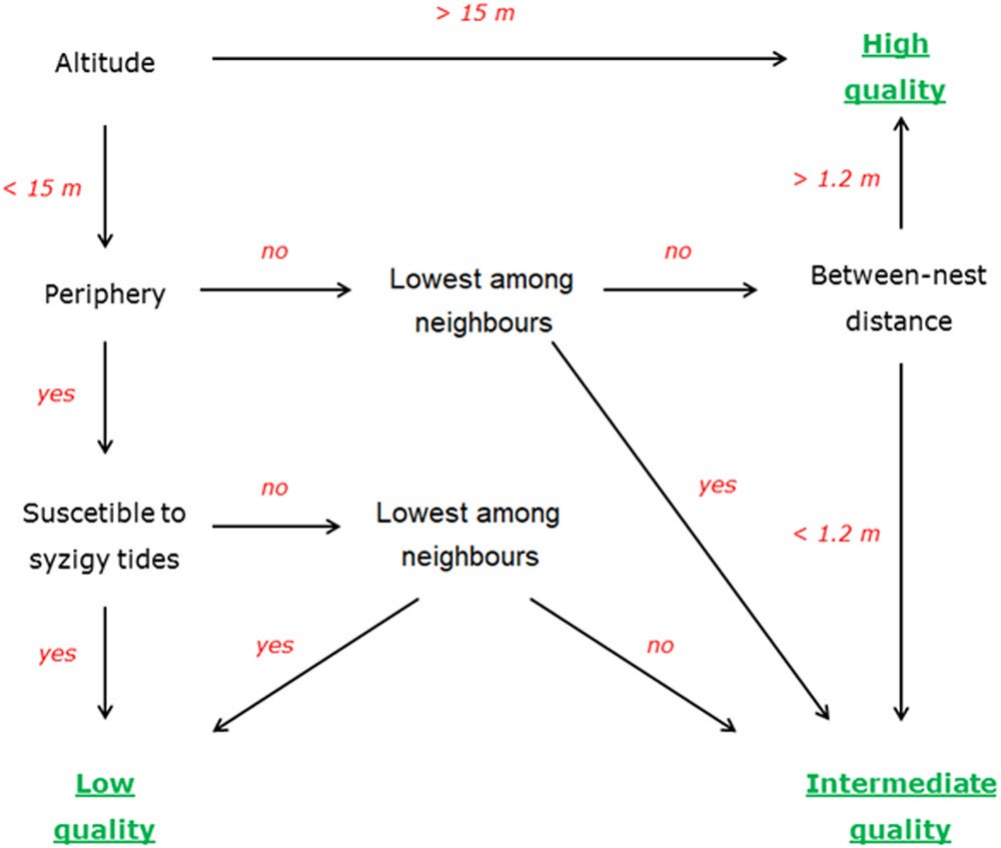
Scheme for classification of nests of brown boobies *Sula leucogaster* breeding in a heterogeneous landscape in the Saint Peter and Saint Paul Archipelago, Brazil, based on the 304 active and non-active nests sampled. A threshold of 15 m a.s.l. was established, so that nests >15 m are protected from waves, even during storms. Peripheral nests were regarded as such when placed within a 3 m-range from the outer limit of the colony. Position in relation to neighbours and between-nest distance were defined by comparing altitude and distance, respectively, of each nest according the three nearest nests. The average between-nest distance for nests <15 m was 1.2 m.

Given the landscape heterogeneity of the colony, the nest quality was determined using a combination of parameters, including global altitude, distance and altitude in relation to their neighbours, distance from the colony edge, and susceptibility to destruction by wave action obtained for all the 304 nests. From this, nests were classified folowing a hierarchical scheme considering the relevance of each metric for quality (Fig. 7). A threshold of 15 m above sea level (a.s.l.) was established as a criterion of quality, because during an extreme event in 2014, all nests below ~15 m were destroyed by waves, representing almost 80% of the active nests of that year (pers. obs.). Altitude is the most important feature in our scheme because nests >15 m are safe even during storms, adults can depart and arrive easily and safely, and nests do not get flooded during raining periods. Hence, nests >15 m were classified as high-quality nests. Colonial breeding has been suggested to be a strategy against predators, and chick survival seems to be higher in central nesting sites^35,61^. Peripheral nests were defined as those within a 3 m-range from the outer limit of the colony. Within peripheral nests, there are some nest locations that are usually destroyed by waves, mainly during syzygy tides. SPSP is primarily influenced by the north branch of the South Equatorial Current, which flows northwestward, and by the southeast trade winds, so that waves predominantly reach the archipelago in its south-southeast portion^60^. However, only the southern part of the colony is exposed to waves, as the east-eastern portion of the colony is protected by the island (Fig. 6). Therefore, nests located in the southern portion of the island were classified as low-quality nests, since they are usually destroyed during syzigy tides. The position relative to neighbouring nests was defined by comparing the altitude of each nest to those of the three nearest nests. This is important because nests in lower places are more susceptible to flooding, soaking eggs and chicks. Nests in the peripheral area with the lowest local altitudes were also classified as low-quality nests. For each nest, distances from the three nearest nests were taken to represent the average between-nest distance. The average between-nest distance for nests <15 m in altitude was 1.2 m (see the ‘Results’ section). Therefore, a nest below the threshold of 15 m was classified as of high quality if it was located outside the peripheral area, it was not at the lowest local altitude among neighbours, and it had an average between-nest distance >1.2 m. All nests that did not fit into the parameters for low or high quality nests were classified as intermediate quality nests.

As an additional approach, each metric was categorized by a binomial score (0: unfavourable, 1: favourable). The five scores were summed up and scaled in order to vary between 0 and 3 (nest quality score (NSQ) = sum of each metric score *3/5). Low quality nests were those with NSQ within [0;1], intermediate quality nests those with NQS within [1;2] and high quality nests those with NSQ within [2;3].

### Sampling scheme

This study was performed in accordance with the Guidelines to the Use of Wild Birds in Research of the Ornithological Council. In addition, all the sampling protocols were approved by The National Center for Bird Conservation and Research (Permit No. 3208) and the Chico Mendes Institute for Biodiversity Conservation (ICMBio Permit No. 38723). During June 2013, May–June 2014 and June–July 2015, morphometrics and body mass measurements from breeding boobies were obtained after capturing individuals on the nests by hand or handnet. Body mass was obtained to the nearest 20 g using Pesola^®^ spring scales, and morphometrics were measured as follows: culmen length (exposed culmen) and tarsus length (from middle of the midtarsal joint to the distal end of the tarsometatarsus) with Vernier calipers (0.01 mm) and wing chord (carpal joint to the tip of the longest primary; unflattened wing) with a metal ruler with stop (1 mm). The gender was determined by differences in the skin coloration around the eyes, and the adults were distinguished from juveniles by plumage coloration^28^. After sampling, each booby was identified with a numbered tarsal metal ring to avoid resampling and aiming monitoring demographic parameters.

Because there is a strong and reverse sexual size dimorphism in brown boobies^28^, during the fieldwork carried out in 2015, boobies for sampling were divided into subgroups based on their body size and each gender was treated as a distinct dataset. To generate a global body size index, the wing chord, culmen and tarsus length, and body mass were standardized by subtracting the mean and dividing by the standard deviation and synthesized through a Principal Component Analysis. The first principal component (PC1), which explained 66.1% of the total variance, was adopted as a body size index and used for sorting individuals. To group the individuals in relation to their body size, males and females PC1 (body size index) were ranked and split into 20% quantiles: the first quantile (0–20%) was treated as the smallest individuals, the third quantile (40–60%) was treated as intermediate individuals, and the fifth quantile (80–100%) was classified as the largest individuals (Supplementary Fig. 5). Because adults in distinct breeding stages were sampled, the non-parametric Kruskal-Wallis test was used to test for the effect of nest content on the foraging behaviour and diet parameters. The chicks were aged as follows: N1 (0–3 weeks); N2 (4–6 weeks); N3 (7–11 weeks); and N4 (12 weeks to fledgling)^62^.

### Foraging behaviour

For studying foraging movements, miniaturized high-resolution GPS units with integrated chip antennae and rechargeable batteries were used (12.5 g, 19 × 25 × 5 mm; Technosmart, Rome, Italy). Loggers were set to record one position every second when deployed for one day and one position every 10 seconds when deployed for two days. During 30 days in July 2015, boobies were captured for logger deployment between 04:00 and 05:00 h, and GPS transmitters were attached to the three central rectrices using TESA tape. Loggers were packaged within heat-shrinkable waterproof tubes, and the total mass of the equipment (*i*.*e*., tube, tape, and logger) did not exceeded 3% of the body mass of the lightest brown booby at SPSP (*i*.*e*., a male of 1185 g), as recommended for seabirds^63^. After logger recovery, data were downloaded using the dedicated GiPSy-4 Utility software (Technosmart, Rome, Italy). Logger deployment and recovery (when biological samples were taken) lasted ~5 and 10 min, respectively, and in both handling procedures, the bird was returned immediately to its nest to minimize stress.

### Diet parameters

Diet of boobies was studied during the same aforementioned period of tracking by analysing stable isotopes of carbon and nitrogen in the blood serum and the regurgitated material. For serum isolation, 3-ml blood samples were taken from the tarsal vein during GPS recovery using a sterile syringe/needle and transferred to a non-heparinized tube, which was kept undisturbed at room temperature for 10–30 min to allow for blood clotting. After clotting, the tubes were centrifuged for 25 min at 3000 RPM to isolate 1 ml of the serum, which was then transferred to 2-ml tubes using clean pipette tips and frozen at −4 °C or lower until processing in the laboratory. Bird serum has an isotopic half-life <5 days^64^ and, thus, the carbon and nitrogen isotopic ratios obtained in this study correspond to the period immediately prior to remote tracking. Spontaneous regurgitation of the stomach content is a usual behaviour in Sulidae species during stressful situations^28^. During handling, the nondigested regurgitated material was collected and identified at species level, and the fork length was measured using a ruler with 1-mm accuracy. Additionally, muscle samples from prey items, which have an isotopic half-life of approximately 30 days for fish, were taken and stored in anhydrous ethanol for stable isotope analysis^65^.

In the laboratory, muscle samples were washed in a Soxhlet extractor during three sessions of 8 h each to remove lipids using a 2:1 chloroform:methanol solution as solvent^66^. Muscle and serum samples were freeze-dried and homogenized, and subsamples of ~0.7 mg were transferred into tin capsules to be analysed using mass spectrometry (measurement precision of 0.2‰) at the Stable Isotope Core, Washington State University (USA). Differences between the sample ratios and the international reference standards (Vienna Pee Dee Belemnite limestone for carbon, and air for nitrogen) were expressed in *δ* notation as parts per thousand (‰)^67^.

### Statistical analyses

The accuracy of GPS positions used for tracking was <10 m in more than 95% of the location fixes, allowing reconstruction of the foraging trips and extraction of high-resolution foraging parameters, such as the total distance travelled (‘D’, km), maximum distance to the colony (‘Dmax’, km), time spent at sea (‘T’, h), sinuosity of the path (‘Sin’, defined as the ratio D.2Dmax^−1^), and average flight speed (‘FS’, km.h^−1^).

Carbon and nitrogen isotopic ratios were used for calculating the isotopic niche width through the Bayesian framework implemented in SIBER package^68^. Standard ellipse areas (‰^2^) corrected for small sample sizes (SEAc) were estimated for each body size category along with the pairwise overlap percentage value between ellipses. Proportions of prey contribution to the diet of each group were estimated with Bayesian mixing models as implemented in MixSIAR package^69^. For this, isotopic ratios of the three most important prey species found in the %PSIRI analysis (see below) were used as source data, and trophic enrichment factors were 1.1 ± 0.5 for *δ*^13^C and 2.8 ± 0.5 for *δ*^15^N, following an experimental study with a piscivorous seabird and lipid-free tissues^70^. Posterior distributions for each prey item for each group were obtained after running 1,000,000 MCMC, discarding the first 500,000 as ‘burn-in’^69^.

Characterization of boobies diet from regurgitated material followed the Prey-Specific Index of Relative Importance (‘%PSIRI’)^71^, which is based on prey-specific parameters (*i*.*e*., ranging from >0% to 100%), such as frequency of occurrence, prey-specific abundance, and prey-specific mass or volume. Because prey may be sampled in various stages of digestion, individual mass was predicted from the fork length by using previously published Bayesian regression parameters for elongated-body fish species^72^.

Differences of boobies body size between the categories of nest quality (considering both approaches to classify nests) were tested with univariate Analysis of Variance (ANOVA), and normality and homoscedasticity of the residuals were tested with Shapiro-Wilk’s and Levene’s tests, respectively. Similarly, the intrasexual differences between the foraging parameters and stable isotopes were tested with the nonparametric Kruskal-Wallis test. As intrasexual relationships between body size and behavioural and diet variables were expected to be non-linear, two distinct approaches were used to test for the association between the isotopic data and foraging trip parameters with PC1. First, intrasexual differences of foraging behaviour and diet were tested with the nonparametric Kruskal-Wallis test by using body size groups as factors. The second approach was based on correlations between the continuous body size index and behavioural and diet variables by using the nonparametric Kendall’s tau (*τ*), which measures the correlation between two ranked variables^73^. Finally, regression trees were used to identify the importance of the body size groups by explaining the variance in each foraging and diet parameter. For classifying variables according to their importance at each regression tree, the Random Forest algorithm implemented in the R package *randomForest* was applied^74^ by generating 1000 regression trees with 20 per-tree permutations for out-of-bag data. The classification procedure was carried out by ranking variables according to the resulting error rate caused when each variable was removed from the model (*i*.*e*., the higher the error rate was, the higher the importance of the variable was).

### Data availability

Data used in this study is deposited in Dryad Digital Repository.

## Electronic supplementary material

Supplementary Information

3D brown boobies colony
