# Supplementary material for: Seabirds fighting for land: phenotypic consequences of breeding area constraints at a small remote archipelago
Source: Sci Rep. 2018 Jan 12;8:665. doi: 10.1038/s41598-017-18808-7 (PMC5766501; doi:10.1038/s41598-017-18808-7)
Supplement: Supplementary file 1 — Supplementary Information [file 41598_2017_18808_MOESM1_ESM.pdf]

Seabirds fighting for land: phenotypic consequences of breeding area constraints at a small remote archipelago

Guilherme Tavares Nunes[1*], Sophie Bertrand[2,3], Leandro Bugoni[1]

[1] Universidade Federal do Rio Grande (FURG), Laboratório de Aves Aquáticas e Tartarugas Marinhas, Instituto de Ciências Biológicas & Programa de Pós-graduação em Oceanografia Biológica, CP 474, Rio Grande, RS, Brazil.

[2] Institut de Recherche pour le Développement (IRD), UMR248 MARBEC, Centre de Recherche Halieutique Méditerranéenne et Tropicale, Avenue Jean Monnet, BP 171, 34203 Sète Cedex, France.

[3] Universidade Federal Rural de Pernambuco (UFRPE), Departamento de Pesca e Aquicultura, Departamento de Biometria, Rua Dom Manuel de Medeiros, s/n, Dois Irmãos, Recife, PE, Brazil.

* Corresponding author: gtn.biomar@yahoo.com.br

This PDF file includes:

Supplementary Table 1

Supplementary Figures 1 to 5

DOI: 10.1038/s41598-017-18808-7

Supplementary Table 1. Carbon and nitrogen isotopic ratios (mean ± 1 standard deviation) from muscle samples of prey items of brown boobies *Sula leucogaster* breeding in the Saint Peter and Saint Paul Archipelago, Brazil, in July 2015.

**Prey species**	**δ^{13}C (‰)**	**δ^{15}N (‰)**
Exocoetus volitans (*n* = 24)	-17.85 ± 0.62	8.66 ± 1.35
E. volitans (large) (*n* = 6)	-17.71 ± 0.56	9.23 ± 1.16
E. volitans (intermediate) (*n* = 13)	-18.10 ± 0.40	7.42 ± 0.61
E. volitans (small) (*n* = 5)	-18.28 ± 0.51	8.98 ± 0.53
Hirundichthys affinis (*n* = 5)	-17.32 ± 0.49	9.83 ± 1.30
Oxyporhamphus micropterus (*n* = 8)	-17.81 ± 0.67	8.90 ± 1.43
Cheilopogon cyanopterus (*n* = 2)	-17.31 ± 0.03	11.11 ± 0.51
Prognichthys gibbifrons (*n* = 6)	-17.59 ± 0.36	9.19 ± 1.19
Euleptorhamphus velox (*n* = 3)	-17.31 ± 0.52	9.68 ± 1.35
Ommastrephes bartramii (*n* = 5)	-17.93 ± 0.27	9.21 ± 0.26

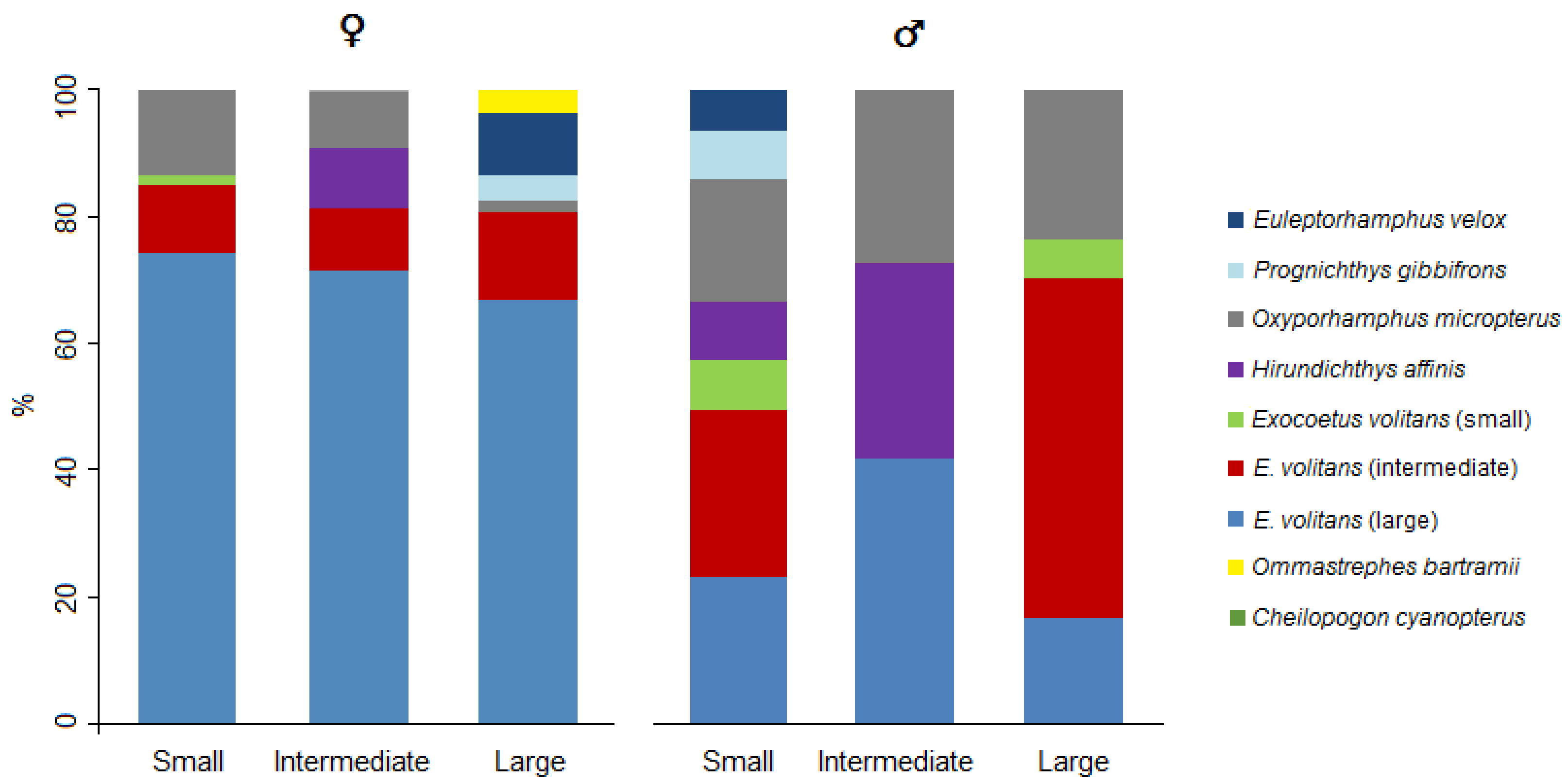

Supplementary Figure 1. Prey-Specific Index of Relative Importance ('%PSIRI'; Brown *et al*. 2012) for each prey item found in regurgitate material of small, intermediate, and large brown boobies *Sula leucogaster* breeding in the Saint Peter and Saint Paul Archipelago, in July 2015. In total, 72 stomach contents and 307 individual prey from 60 distinct individual birds (36 females and 24 males) were analyzed. The asymptote of prey species richness was reached with 21 stomach contents and 55 prey. Due to its high prey-specific importance, *E. volitans* were separated into three food item categories according to fork length: small (< 100 mm), intermediate (100−150 mm) and large individuals (> 150 mm).

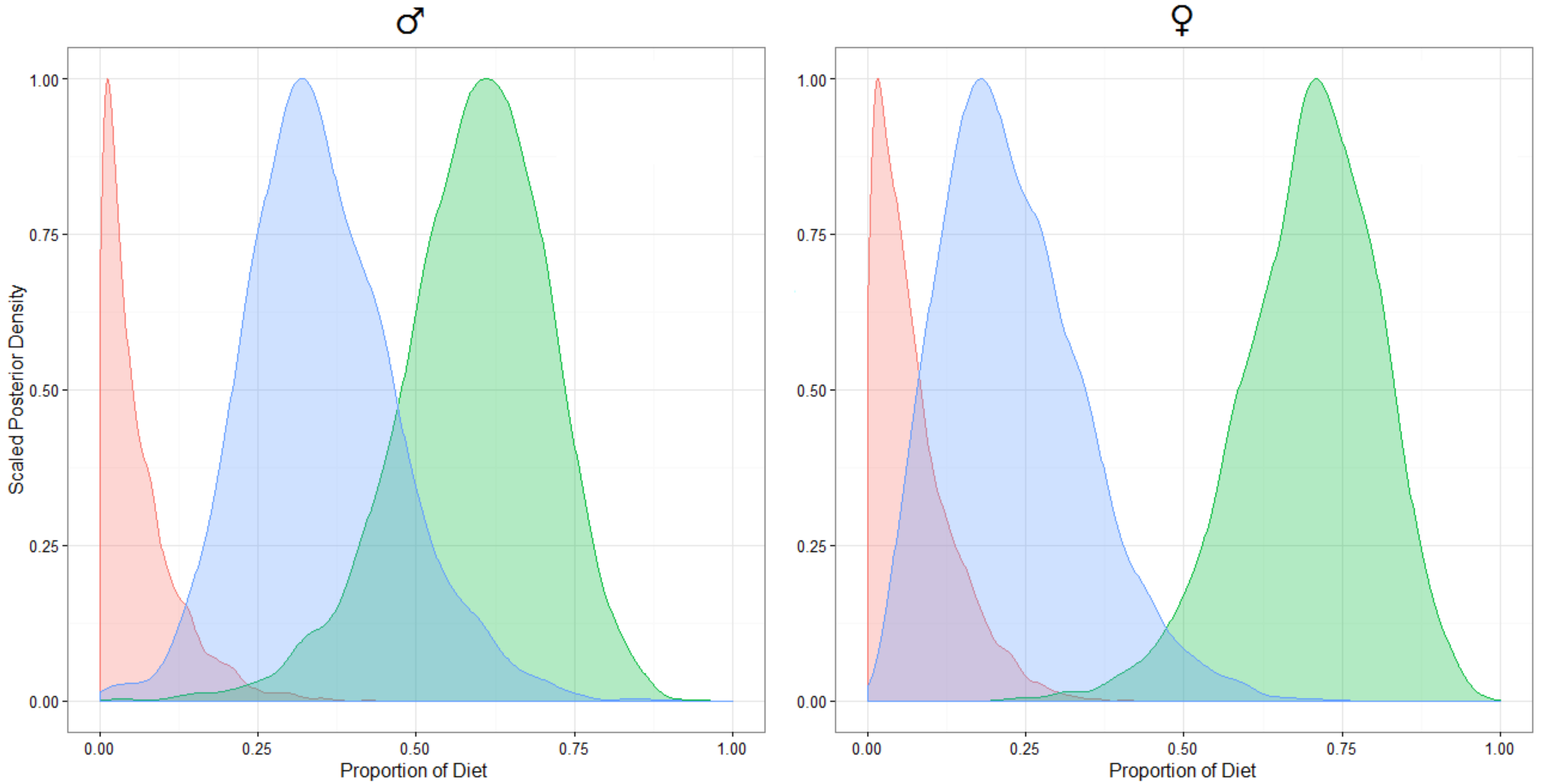

Supplementary Figure 2. Posterior probabilities with 95% credibility intervals of intermediate *Exocoetus volitans* (red), large *E. volitans* (green), and *Oxyporhamphus micropterus* (blue) used by female and male brown boobies in the Saint Peter and Saint Paul Archipelago (July 2015), estimated with Bayesian mixing models with carbon and nitrogen isotopic ratios obtained from muscle samples of prey and blood serum of brown boobies.

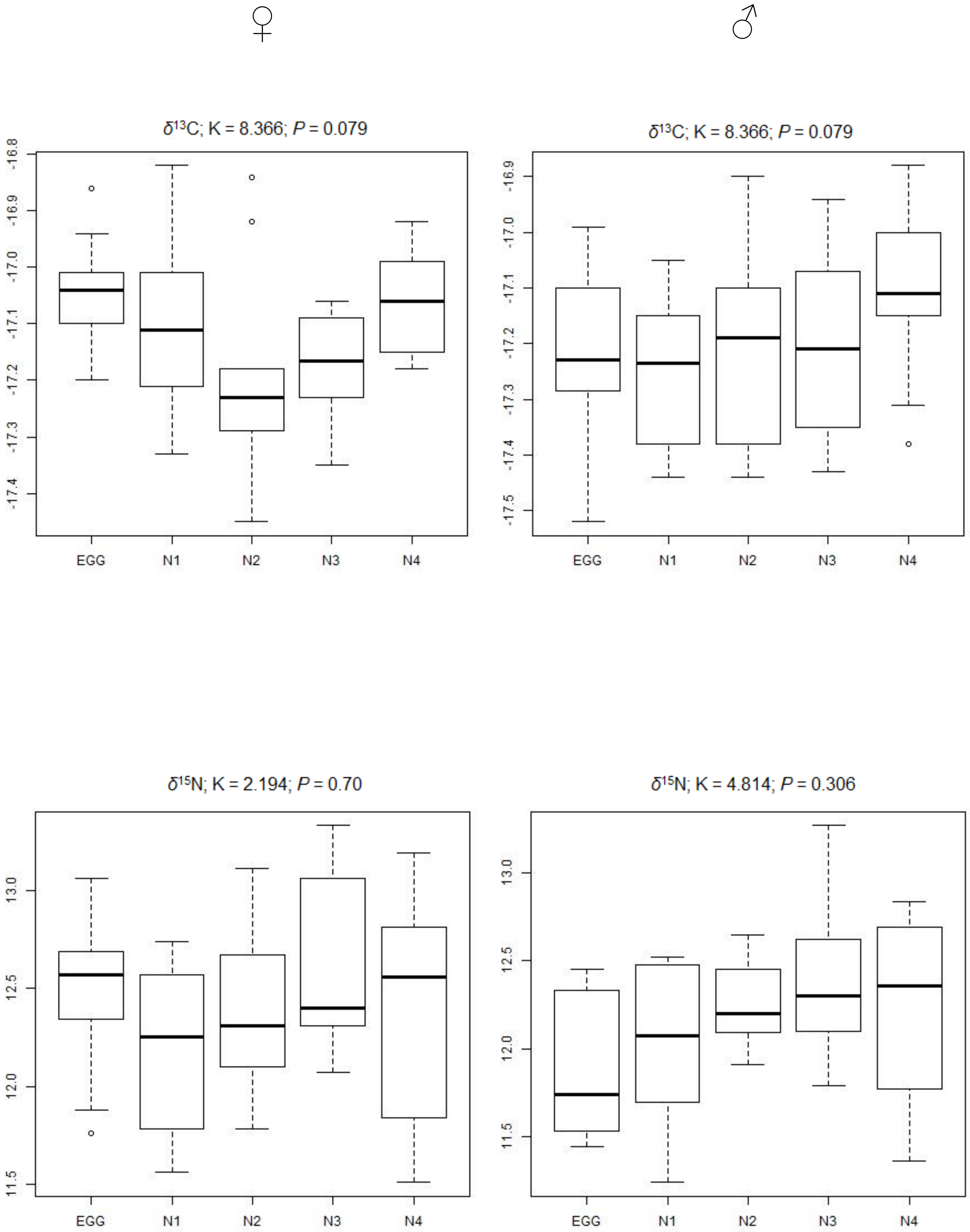
♀
♂
δ13C; K = 8.366; P = 0.079
δ13C; K = 8.366; P = 0.079
δ15N; K = 2.194; P = 0.70
δ15N; K = 4.814; P = 0.306
EGG
N1
N2
N3
N4

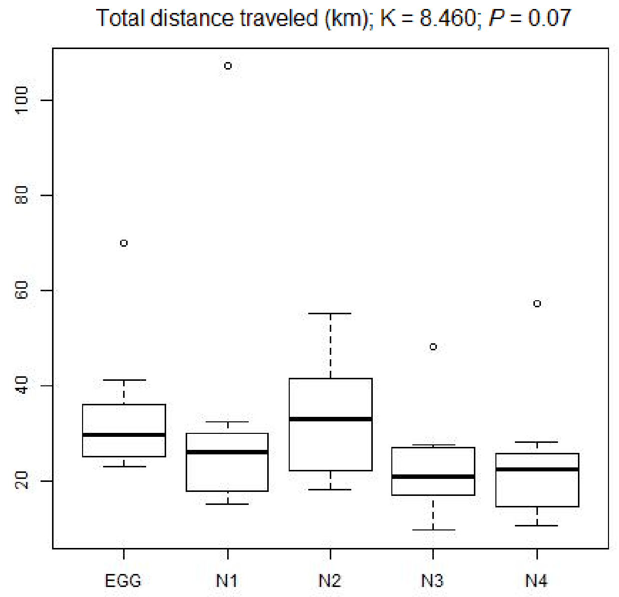
Total distance traveled (km); K = 8.460; *P* = 0.07
20
40
60
80
100
EGG
N1
N2
N3
N4

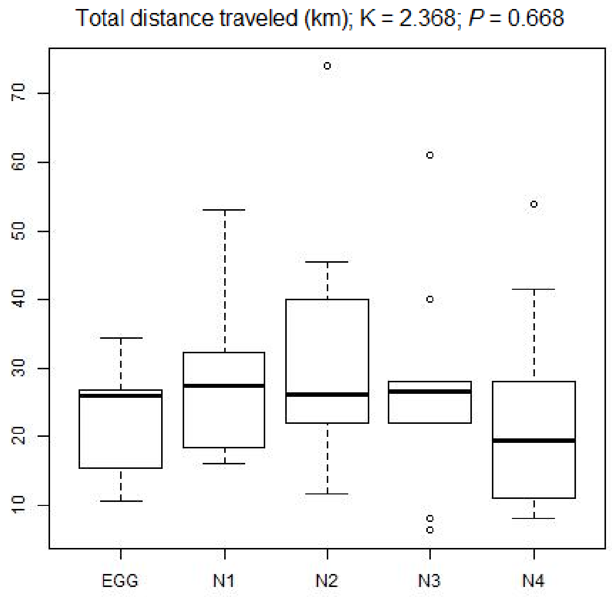
Total distance traveled (km); K = 2.368; *P* = 0.668
10
20
30
40
50
60
70
EGG
N1
N2
N3
N4

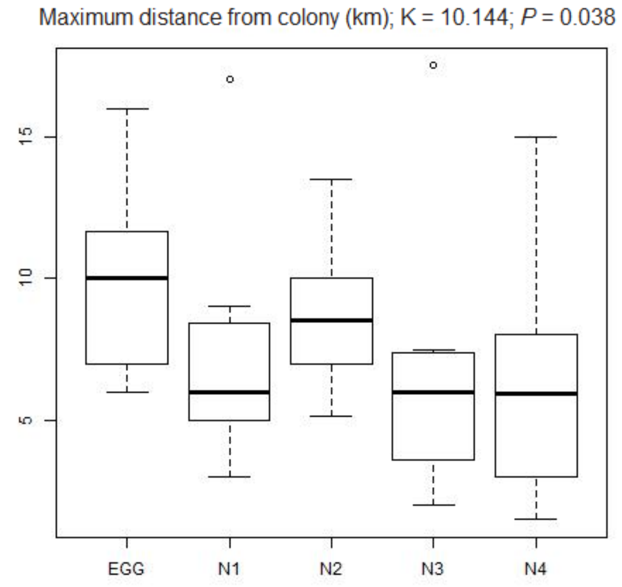
Maximum distance from colony (km); K = 10.144; *P* = 0.038
5
10
15
EGG
N1
N2
N3
N4

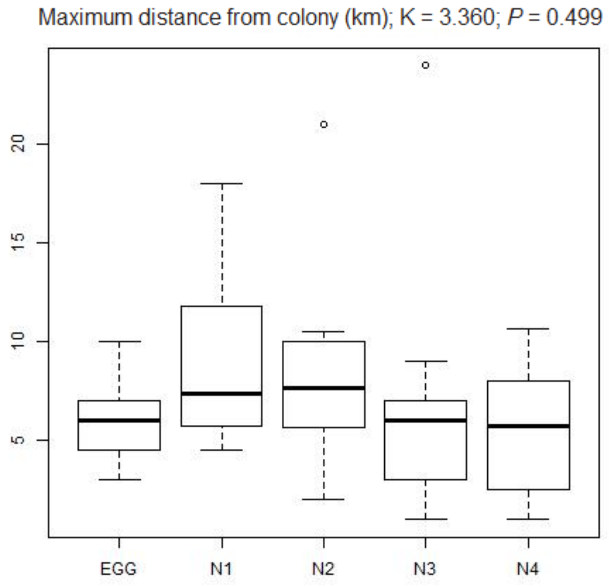
Maximum distance from colony (km); K = 3.360; *P* = 0.499
5
10
15
20
EGG
N1
N2
N3
N4

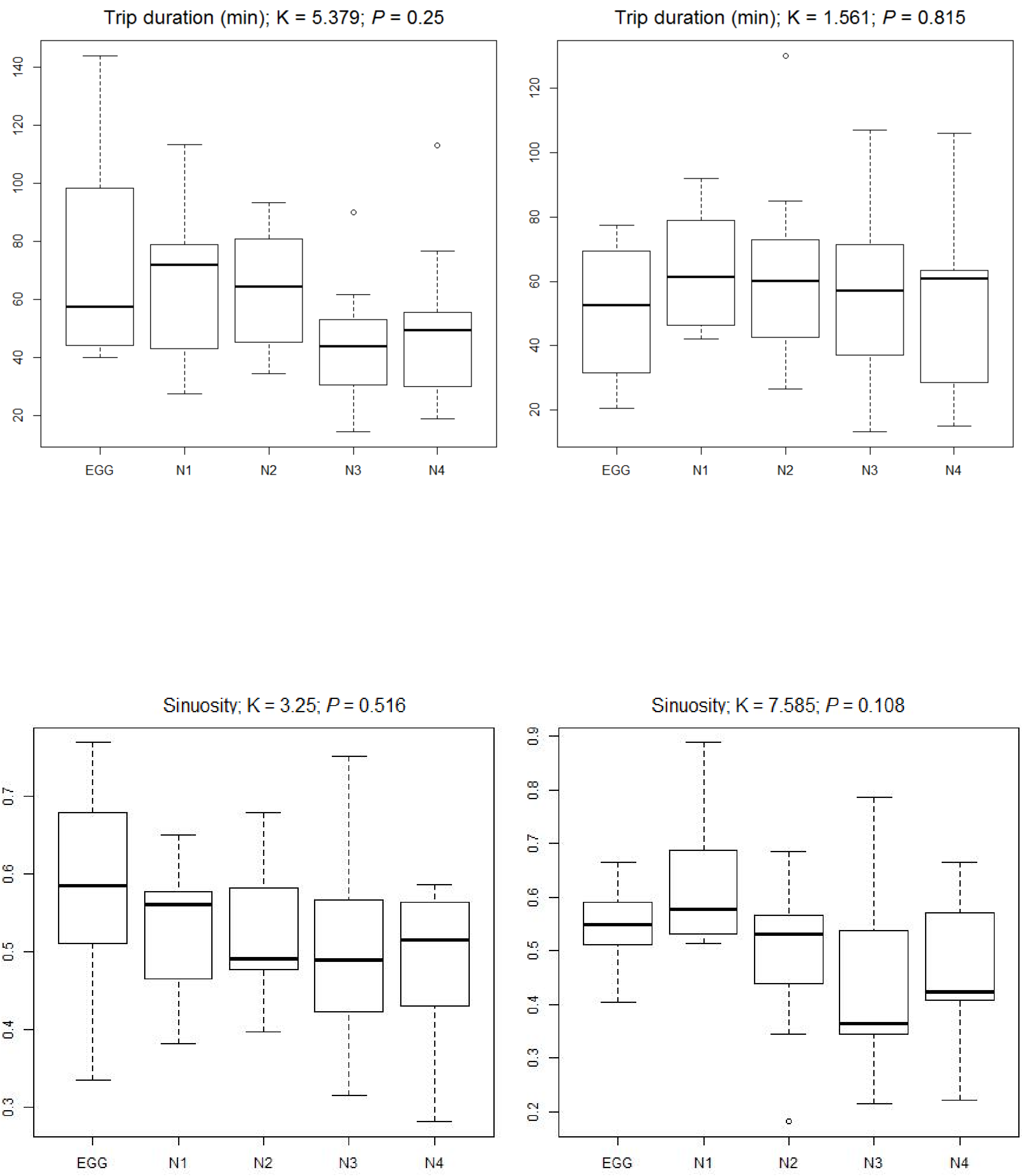

Supplementary Figure 3. Comparisons of diet and foraging behaviour parameters (indicated on top of each figure) between brown boobies *Sula leucogaster* in distinct breeding stages in the Saint Peter and Saint Paul Archipelago, in July 2015. The non-parametric Kruskal-Wallis test (K) was performed to assess significant differences between breeding stages. Chicks were aged using the classification suggested by Simmons (1967): N1 (0–3 weeks); N2 (4–6 weeks); N3 (7–11 weeks); N4 (12 weeks to fledgling).

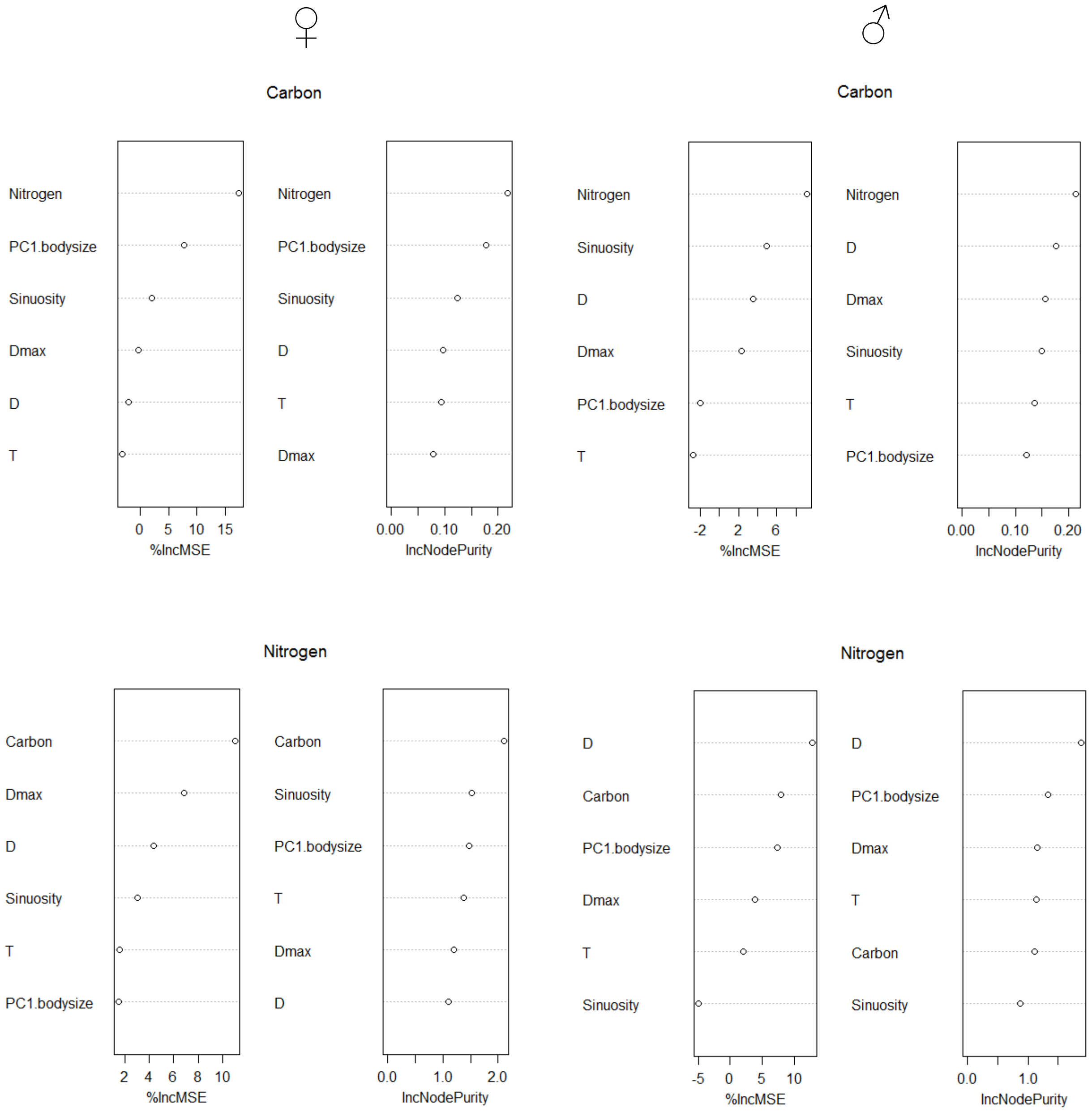

♀
Carbon
Nitrogen
PC1.bodysize
Sinuosity
Dmax
D
T
0 5 10 15
%IncMSE
Nitrogen
PC1.bodysize
Sinuosity
D
T
Dmax
0.00 0.10 0.20
IncNodePurity
♂
Carbon
Nitrogen
Sinuosity
D
Dmax
PC1.bodysize
T
-2 2 6
%IncMSE
Nitrogen
D
Dmax
Sinuosity
T
PC1.bodysize
0.00 0.10 0.20
IncNodePurity
Nitrogen
Carbon
Dmax
D
Sinuosity
T
PC1.bodysize
2 4 6 8 10
%IncMSE
Carbon
Sinuosity
PC1.bodysize
T
Dmax
D
0.0 1.0 2.0
IncNodePurity
Nitrogen
D
Carbon
PC1.bodysize
Dmax
T
Sinuosity
-5 0 5 10
%IncMSE
D
PC1.bodysize
Dmax
T
Carbon
Sinuosity
0.0 1.0
IncNodePurity

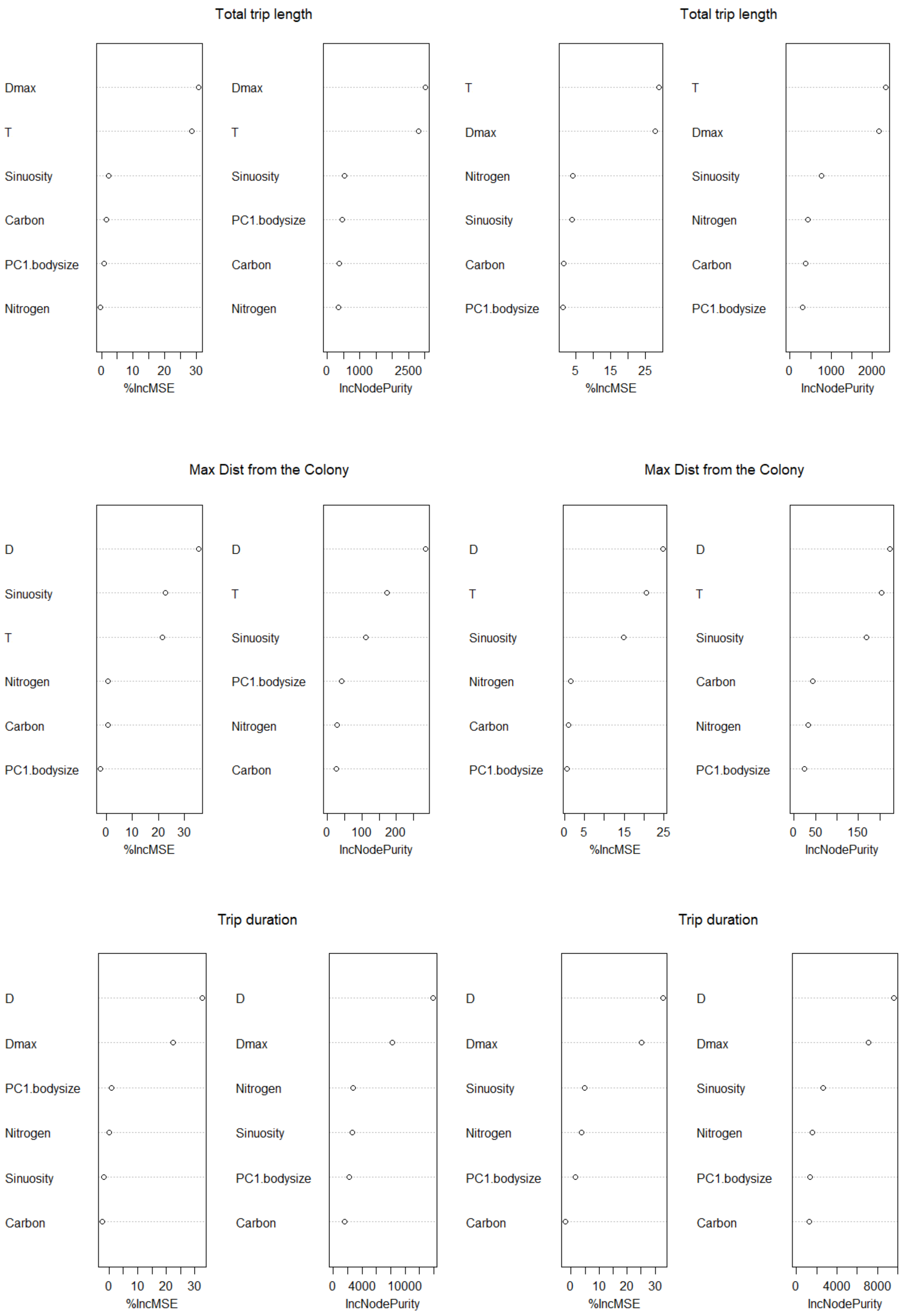

Total trip length
Dmax
T
Sinuosity
Carbon
PC1.bodysize
Nitrogen
0 10 20 30
%IncMSE
Dmax
T
Sinuosity
PC1.bodysize
Carbon
Nitrogen
0 1000 2500
IncNodePurity
Total trip length
T
Dmax
Nitrogen
Sinuosity
Carbon
PC1.bodysize
5 15 25
%IncMSE
T
Dmax
Sinuosity
Nitrogen
Carbon
PC1.bodysize
0 1000 2000
IncNodePurity
Max Dist from the Colony
D
Sinuosity
T
Nitrogen
Carbon
PC1.bodysize
0 10 20 30
%IncMSE
D
T
Sinuosity
PC1.bodysize
Nitrogen
Carbon
0 100 200
IncNodePurity
Max Dist from the Colony
D
T
Sinuosity
Nitrogen
Carbon
PC1.bodysize
0 5 15 25
%IncMSE
D
T
Sinuosity
Carbon
Nitrogen
PC1.bodysize
0 50 150
IncNodePurity
Trip duration
D
Dmax
PC1.bodysize
Nitrogen
Sinuosity
Carbon
0 10 20 30
%IncMSE
D
Dmax
Nitrogen
Sinuosity
PC1.bodysize
Carbon
0 4000 10000
IncNodePurity
Trip duration
D
Dmax
Sinuosity
Nitrogen
PC1.bodysize
Carbon
0 10 20 30
%IncMSE
D
Dmax
Sinuosity
Nitrogen
PC1.bodysize
Carbon
0 4000 8000
IncNodePurity

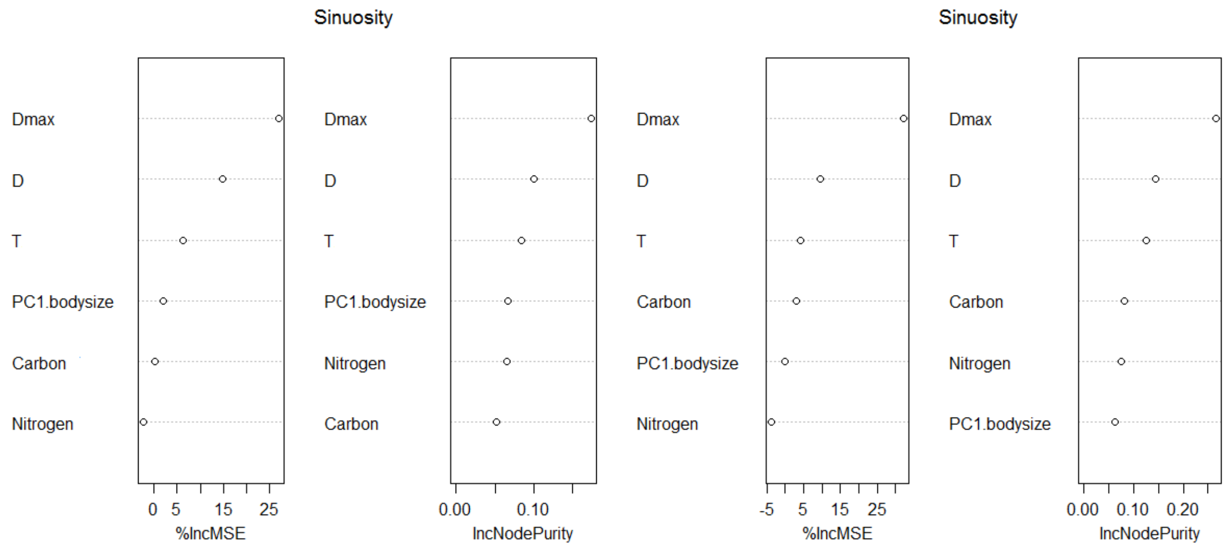

Supplementary Figure 4. Importance of dependent variables in models performed with 1000 regression trees and 20 per tree permutations for out-of-bag data, through the Random Forest algorithm. %IncMSE represents how worse the model performs without each variable, so that very predictive and important variables are characterized by a high decrease in accuracy (variables at the top). IncNodePurity measures how pure the nodes are at the end of the tree. Carbon = δ^{13}C; Nitrogen = δ^{15}N; D = mean total distance ('Total trip length'); Dmax = mean maximum distance from colony ('Max Dist from the Colony'); T = trip duration; Sinuosity = Dist.2Dmax^{-1}; PC1.bodysize = body size index composed by culmen length, tarsus length, wing chord, and body mass data converted in a principal component (PC1).

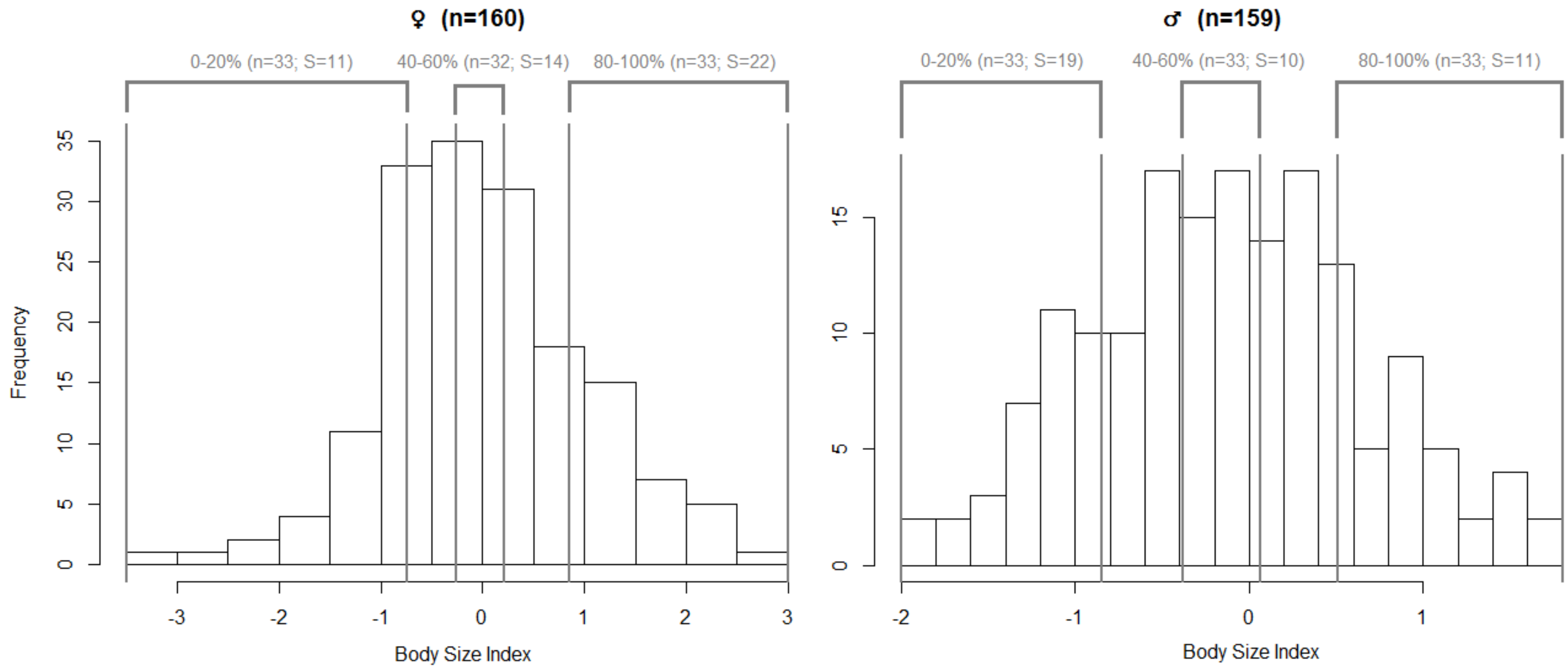

Supplementary Figure 5. Distribution of the Body Size Index for female and male brown boobies *Sula leucogaster* breeding in the Saint Peter and Saint Paul Archipelago, Brazil. Body Size Index represents culmen length, tarsus length, wing chord, and body mass data converted in a principal component (PC1), which explained 66.1% of the total variance. Individuals were divided into body size groups by defining cutpoints between quantiles of 20%, so that the first quantile (0–20%) was treated as the smallest individuals, the third quantile (40–60%) was treated as intermediate individuals, and the fifth quantile (80–100%) was classified as the largest individuals. 'n' represents the number of individuals in each group, and 'S' represents the number of individuals sampled for diet and foraging behavior parameters.
